# Local Fixation of Colistin With Fibrin Spray: An *in vivo* Animal Study for the Therapy of Skin and Soft Tissue Infections

**DOI:** 10.3389/fsurg.2022.749600

**Published:** 2022-03-17

**Authors:** Maren Janko, Fabian Dust, Pia Viktoria Wagner, Robert Gurke, Johannes Frank, Dirk Henrich, Ingo Marzi, René Danilo Verboket

**Affiliations:** ^1^Department of Trauma-, Hand and Reconstructive Surgery, Hospital of the Goethe-University, Goethe-University, Frankfurt, Germany; ^2^Fraunhofer Institute for Translational Medicine and Pharmacology ITMP, Frankfurt, Germany; ^3^Pharmazentrumfrankfurt/ZAFES, Department of Clinical Pharmacology, Johann Wolfgang Goethe University, Frankfurt, Germany

**Keywords:** acute infections, chronic infections, soft tissue, SSTI, local antibiotics, fibrin-glue-spray technique, animal model

## Abstract

**Objective:**

Skin and soft tissue infections (SSTI) are a commonly known entity of diseases associated with difficult treatment procedures. The current gold standard when there is a rapidly progressing infection of soft tissues with a risk of sepsis is radical surgical debridement accompanied by systemic antibiotic therapy. In clinical settings, local antibiotics alone or formulated within carrier material are commonly used alongside this therapy regimen. One possibility of local antibiotic application is the fixation of colistin with fibrin glue spray. It is not yet sufficiently researched how the local antibiotic concentrations remain as high as possible over time.

**Methods:**

We conducted an animal study including 29 male Wistar rats inducing sterile back sores reaching the muscle fascia. We sprayed only colistin, simultaneously or consecutively, with fibrin glue in different groups in order to measure the tissue concentration of the antibiotic applied locally.

**Results:**

After liquid chromatography and quadrupole mass spectrometry analysis, it could be demonstrated that in comparison to the colistin group, tissue concentrations of colistin stayed significantly higher in the wound tissue when it was fixed with fibrin glue. This was observed in both groups, the simultaneous as well as in the consecutively fibrin glue sprayed groups after colistin application.

**Conclusion:**

The fixation of colistin with the fibrin-glue-spray technique as a carrier for local antibiotic therapy is an easy and inexpensive method and shows promising potential for the treatment of SSTI.

## Introduction

Skin and soft tissue infections (SSTI) are a heterogeneous group of diseases characterized by microbial colonization of all skin layers as well as the subdermal connective tissue layers (1). SSTI can lead to infection of the bone (osteitis) and to infection of the marrow of the bone (osteomyelitis) (2). Infection of the skin and soft tissue is for the most part owing to an exogenous cause. There is either traumatic or surgical contamination. As a result, there may be a complex immune response between the infecting microorganisms and the host (3).

The treatment of chronic SSTI is often a lengthy, expensive and problematic undertaking (4, 5). The pillars of the treatment consist of infection control by surgical debridement and pathogen-specific antibiotic therapy, soft tissue coverage and restoration of bone integrity (5). Infection with multi-resistant pathogens further complicates the therapy.

The gold standard in the treatment of soft tissue and bone infection is still surgical debridement, which in its radicality is oriented toward tumor surgery (5). Here, the close interlocking of bone and SSTI, which are mutually dependent in their physiopathogenesis, becomes clear. Both the soft tissue debridement and the bone debridement must be carried out generously, complete and in sterile conditions, on the one hand to prevent a re-emergence of the infection and on the other hand, a greater tissue loss in case of progressive infection and the resulting dead space should be contained (6). The resulting avascular and bradytroph space due to scarring is not difficult to achieve for immunocompetent cells. Additionally, systemically applied antibiotics reach it only in small amounts due to the avascularity of the dead space and cannot build up effective concentrations locally. It is therefore important to cover the dead space with locally active antibiotic agents (7).

In addition to standard antibiotic therapy, i.e., systemic antibiotic administration, Buchholz et al. (8) in 1970 developed the method of using bone cement mixed with antibiotics for hip joint implantation. Bone cement based on polymerized polymethyl methacrylate (PMMA), usually in combination with the aminoglycoside antibiotic gentamycin, is still by far the most widely used material for local antibiotic therapy (9). In addition, there are adhesive carrier substances with an incorporated antibiotic on the market, e.g., a collagen gentamycin sponge, diverse antibiotic-coated osteosynthesis materials, biodegradable gels with antibiotic content, antibiotics in powder form and calcium compounds with antibiotic active agents.

A new possibility of local antibiotic application is the spraying of the wound surface with a mixture of antibiotic and fibrin glue. The effective antibiotic agent determined after resistance testing is applied to the wound bed in combination with the fibrin glue by a sprayer and fixed locally there. Fibrin glue consists of two components: a fibrin glue protein, which contains human fibrinogen as the main ingredient, and human thrombin. When both components come into contact, fibrin, the final product of plasmatic clotting, is produced. In a retrospective study by Janko et al. (10), 21 patients with acute or chronic bone infection and concomitant SSTI were treated with a combination of antibiotic and fibrin glue. After spraying with the mixture of antibiotic and fibrin glue, eight out of nine areas of these severely infected extremities treated could be brought to cure.

The study by Janko et al. thus presented another type of local use of antibiotics in bony infections, which places greater emphasis on the aspect of soft tissue participation (10). The questions derived from this clinical study about tissue effect, length of stay, systemic absorption and best method of application of antibiotics were then evaluated in an animal study by Verboket et al. (11). The concentration of the glycopeptide antibiotic vancomycin in tissue samples was measured after 1, 2, and 4 h, which was applied with a sprayer to soft tissue wounds on the back of 39 Sprague-Dawley rats. The interpretation of the study results allows the statement that the local drug concentration of vancomycin by fibrin fixation over a period of 4 h is higher than the active substance concentration of vancomycin without fibrin fixation. The fibrin fixation of vancomycin is rated as a benefit.

## Materials and Methods

### Animal Care

All animal tests took place under applicable standards and after official approval by the Darmstadt Regional Council (project number Gen. FK/1,133). A total of 29 male rats from the strain *Wistar* (Envigo RMS GmbH, Roßdorf, Germany) with a weight of 330–365 g were in cages with three to five animals in each and received food and water *ad libitum*. At the time of the experiment, the animals were 8 weeks old. The cages were housed in constantly ventilated, temperature- (15–21°C) and light- (14 h day: 10 h night) controlled rooms of the Central Research Institute (ZFE) of Goethe University Frankfurt am Main (Sandhofer Allee, Frankfurt am Main, Germany).

### Preparation of the Colistin/Fibrin Mixture

In the experiment, one million I.E. colistimethate sodium (CMS) in powder form *(Promixin*, Zambon, Berlin, Germany) were dissolved in 20 ml of 0.9% isotonic NaCl solution (Braun, Melsungen, Germany) and put in a 10 ml syringe. One million I.E. CMS corresponds to ~80 mg of CMS and 33.3 mg colistin.

### Surgery and Wound Spraying

The rats were anesthetized by intraperitoneal administration of Ketaset 100 mg/ml (Zoetis, New Jersey, USA) and Xylavet 20 mg/ml (CP-Pharma, Burgdorf, Germany) in weight-adjusted doses. The intraperitoneally applied injection volume was 1–2.5 ml. The back of the rat was shaved, cleaned and disinfected with Octeniderm (Schülke, Norderstedt, Germany). After sterile covering of the shaved and disinfected surface with a sterile perforated cloth, four wounds, each 100 mm^2^, were placed under aseptic conditions by means of scalpel and scissors, reaching the muscle/fascia level. The fascia was roughened with the scalpel. This was followed by the spraying of the wounds according to the group. After the operation, the animals were placed individually and after 1, 2, and 4 h, depending on the group, killed by an overdose of pentobarbital 500 mg/kg i.p. and the addition of two-sided pneumothoraxes. Cardiac blood and tissue samples were taken from the wound surfaces and the blood, transferred to EDTA tubes (blood) and 1 ml of Eppendorf tubs (tissue samples) and stored in the laboratory on dry ice at −80°C until sample preparation and measurement.

The two pre-filled syringes of the fibrin glue *TISSUCOL DUO S Immuno*^©^ were used as described in the *DUPLOJECT*^©^ syringe attachment and connected to the *EASYSPRAY*^©^ pressure regulator. The distance from the wound to the spray head was 10–15 cm and the *EASYSPRAY*^©^ pressure regulator was adjusted to the recommended spray pressure of 1.5–2.0 bar (21.5–28.5 psi). To prevent cross-contamination, the back wounds of a single animal were treated exclusively by one of the three possible types of application. Thus, a possible systemic uptake of the antibiotic or its enrichment in the remaining back wounds was prevented. Blood samples were taken to monitor a systemic uptake. The groups used in the experiment were shown in Table 1.

**Table 1:** Groups, timepoints and number of samples used in the experiment.

	**1 h**	**2 h**	**4 h**
Simultaneous spraying of colistin and fibrin glue (CF+)	*n* = 7	*n* = 7	*n* = 7
Consecutive spraying of colistin and fibrin glue (CF-)	*n* = 7	*n* = 7	*n* = 7
Without fixation of colistin by fibrin glue (C)	*n* = 7	*n* = 7	*n* = 7

### LC-MS/MS-Analysis

Colistin sulfate (polymyxin E; colistin A 31.1%; colistin B 53.6%; Sigma Aldrich, Steinheim, Germany) as well as polymyxin B (Merck, Darmstadt, Germany) as an internal standard were used. The following solvents were provided by Carl Roth (Karlsruhe, Germany): water (LC-MS-grade), isopropyl alcohol (purity ≥ 99.95%), methanol (purity ≥ 99.95) and acetonitrile (purity ≥ 99.95%). Formic acid (purity 99–100%) was used from VWR Prolabo chemicals (Darmstadt, Germany) and ethanol (purity ≥ 99.8%) from Sigma Aldrich (Steinheim, Germany).

The muscle samples were weighed and a weight-dependent water volume was added. The samples were then homogenized under cooling (Precellys 24^®^, VWR, Darmstadt Germany) to generate a final tissue concentration of 0.075 mg/l. Twenty microliter internal standard (polymixin B, 63 g/ml in water) and 300 μl methanol were added to 125 μl homogenate. After vortexing and centrifuging (5 min, 20,000 g), the supernatant was evaporated under a gentle flow of nitrogen at 45°C temperature and resuspended with 100 μl 0.1% formic acid. Standards and quality control samples were correspondingly tested with homogenized raw matrix (mice muscle tissue without injected antibiotics) and the corresponding standard (colistin A 3.11–3,110 ng: colistin B 5.36–5,360 ng in water).

The LC-MS/MS analysis was performed with an Agilent 1,290 Infinity LC system with binary HPLC pump, column furnace and autosampler (Agilent, Waldbronn, Germany) in conjunction with a triple quadrupole mass spectrometer QTRAP 5500 (AB Sciex, Darmstadt, Germany). A Luna^®^ Omega Polar C18 column (100 × 2.1 mm, 1.6 m, Phenomenex, Aschaffenburg, Germany) was used with a flow rate of 0.3 ml/min and a column temperature of 40°C. Solvents were 0.5% formic acid (solvent A) and acetonitrile/isopropyl alcohol/acetone (v:v:v 5:3:2) with 1% formic acid (solvent B) with the following gradient (time, percentage B): 0.0 min, 2%; 1.0 min, 2%; 6.0 min, 100%; 7.0 min, 100%; 7.5 min, 2%; 10.0 min, 2%. Ten microliter of each sample were injected. The data were collected with Analyst Software 1.7.1 and the quantification was carried out with MultiQuant Software 3.0.3 (both Sciex, Darmstadt, Germany) using the internal standard method. Calibration curves were calculated by linear regression with 1/x weight.

### Statistics

The primary investigation variable is the concentration of the antibiotic after 1, 2, and 4 h. The number of samples per group was *n* = 7. The results are shown in a box plot. To distinguish the significant characteristics of the results of the individual groups, a Kruskal-Wallis test was carried out with multiple Conover-Iman comparisons for *post-hoc* analysis. A *p* < 0.05 (Bonferroni-Holm-corrected) is considered significant. The software used is *BiAS for Windows* version 11.12 dated 01/2021.

## Results

### After 1 H

In the group with simultaneous spraying of colistin and fibrin glue (CF+), the median concentration of colistin in the tissue sample after 1 h was 185.67 ng/mg (standard deviation 0 ng/mg, minimum 185.67 ng/mg, maximum 185.67 ng/mg, range 0 ng/mg, 1st quartile 185.67 ng/mg, 3rd quartile 185.67 ng/mg, interquartile range 0 ng/mg).

In the group with consecutive spraying of colistin and fibrin glue (CF-), the median concentration of colistin in the tissue sample after 1 h was 89.45 ng/mg (standard deviation 29.2671 ng/mg, minimum 55.36 ng/mg, maximum 140.79 ng/mg, range 85.43 ng/mg, 1st quartile 62.11 ng/mg, 3rd quartile 113.45 ng/mg, interquartile range 51.34 ng/mg).

**Figure 1:**
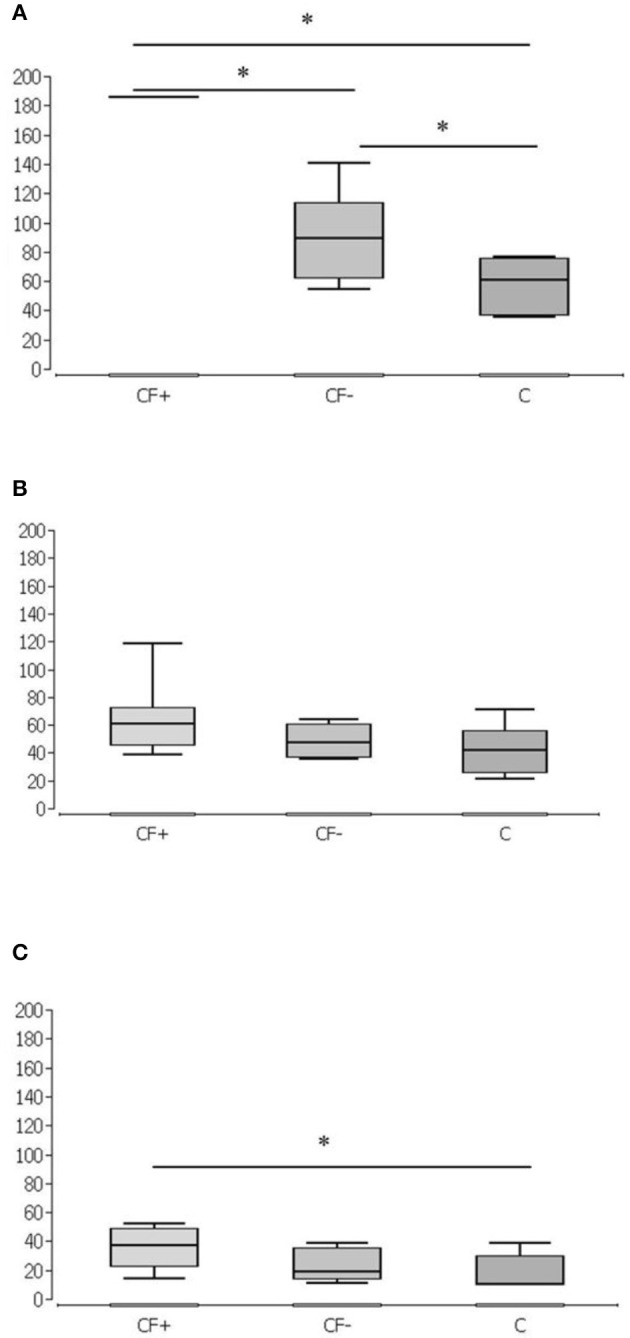
Different spraying techniques of Colistin over time. Simultaneous spraying of colistin and fibrin glue (CF+), consecutive spraying of colistin and fibrin glue (CF-) and without fixation of colistin by fibrin glue (C) after 1 h **(A)**, 2 h **(B)**, and 4 h **(C)**. Significantly higher values in CF+ group at 1 h against CF- and C and in CF- against C **(A)**. No significant differences after 2 h **(B)**. Significantly higher values in CF+ at 4 h against C (*n* = 7 per group and timepoint). *p < 0.05.

In the group without fixation of colistin by fibrin glue (C), the median concentration of colistin in the tissue sample after 1 h was 60.95 ng/mg (standard deviation 16.6957 ng/mg, minimum 36.01 ng/mg, maximum 67.58 ng/mg, range 40.57 ng/mg, 1st quartile 37.24 ng/mg, 3rd quartile 76.03 ng/mg, interquartile range 40.57 ng/mg). In all blood samples, no colistin was detected (Figure 1A).

### After 2 H

In the group with simultaneous spraying of colistin and fibrin glue (CF+), the median concentration of colistin in the tissue sample was 61.19 ng/mg after 2 h (standard deviation 26.4977 ng/mg, minimum 39.4 ng/mg, maximum 118.36 ng/mg, range 78.96 ng/mg, 1st quartile 46.18 ng/mg, 3rd quartile 72.61 ng/mg, interquartile range 87.96 ng/mg).

In the group with consecutive spraying of colistin and fibrin glue (CF-), the median concentration of colistin in the tissue sample after 2 h was 47.47 ng/mg (standard deviation 10.6234 ng/mg, minimum 36.52 ng/mg, maximum 64.03 ng/mg, range 27.51 ng/mg, 1st quartile 36.9 ng/mg, 3rd quartile 60.64 ng/mg, interquartile range 23.74 ng/mg).

In the group without fixation of colistin by fibrin glue (C), the median concentration of colistin in the tissue sample was 17.9217 ng/mg after 2 h (standard deviation 17.663 ng/mg, minimum 21.93 ng/mg, maximum 71.47 ng/mg, range 49.54 ng/mg, 1st quartile 25.96 ng/mg, 3rd quartile 55.90 ng/mg, interquartile range 29.94 ng/mg) (Figure 1B). Additionally, after 2 h, no colistin was detected in the blood samples.

### After 4 H

In the group with simultaneous spraying of colistin and fibrin glue (CF+), the median concentration of colistin in the tissue sample after 4 h was 37.00 ng/mg (standard deviation 13.9312 ng/mg, minimum 14.96 ng/mg, maximum 52.44 ng/mg, range 37.48 ng/mg, 1st quartile 22.78 ng/mg, 3rd quartile 49.34 ng/mg, interquartile range 26.56 ng/mg).

In the group with consecutive spraying of colistin and fibrin glue (CF-), the median concentration of colistin in the tissue sample was 19.03 ng/mg after 4 h (standard deviation 10.7507 ng/mg, minimum 11.84 ng/mg, maximum 38.40 ng/mg, range 26.84 ng/mg, 1st quartile 13.96 ng/mg, 3rd quartile 35.48 ng/mg, interquartile range 35.48 ng/mg).

In the group without fixation of colistin by fibrin glue (C), the median concentration of colistin in the tissue sample was 9.95 ng/mg after 4 h (standard deviation 12.1794 ng/mg, minimum 9.95 ng/mg, maximum 38.78 ng/mg, range 28.83 ng/mg, 1st quartile 9.95 ng/mg, 3rd quartile 29.93 ng/mg, interquartile range 19.98 ng/mg) (Figure 1C). After 4 h, no colistin was detected in the blood samples.

**Figure 2:**
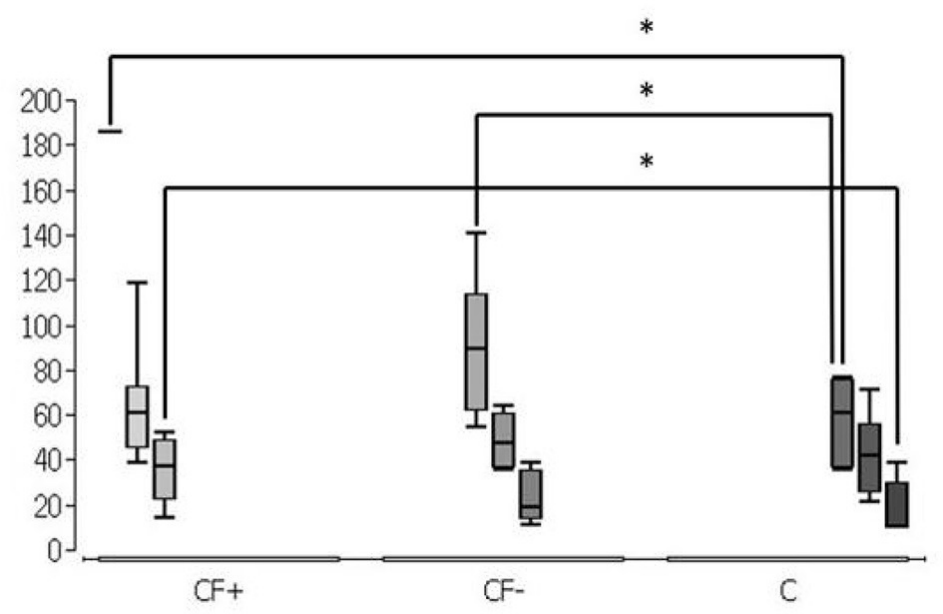
Grouped representation of the respective application methods, combined representation. Simultaneous spraying of colistin and fibrin glue (CF+), consecutive spraying of colistin and fibrin glue (CF-) and without fixation of colistin by fibrin glue (C). Significantly higher values in CF+ group at 1 h against CF- and C and in CF- against C. Furthermore, significantly higher values in CF+ at 4 h against C (*n* = 7 per group and timepoint). *p < 0.05.

Group comparison over time is illustrated in Figure 2, significantly higher colistin values were measured in the CF+ group and CF- group at 1 h vs. the C group. At 4 h significantly higher colistin values were measured in the CF+ group vs. the C group (Figure 2).

## Discussion

The tissue concentration of colistin in an *in vivo* wound model after fibrin spraying was investigated. The fixation of the antibiotic with fibrin glue is a possibility for local antibiotic therapy in bone and SSTI or their prophylaxis, because simple application of soluble antibiotics on an wound surface usually holds very short time (10, 11).

Post-traumatic or postoperative bone infections with soft tissue involvement are serious complications that are treated by surgical therapy and adjuvant intravenous antibiotic therapy or by intravenous antibiotic therapy alone. The standard antibiotic therapy for bone and SSTI is systemic antibiotic administration (12). However, sometimes this is not enough (4). Prosthetic-associated infections are difficult to treat systemically with antibiotics, as the causal bacterial strains form a biofilm and systemically applied antibiotics penetrate it poorly. In these cases, careful consideration must be given in conjunction with the patient's other conditions. In some cases, preservation of the prosthesis is possible with intravenous or local antibiotic therapy. In other cases, the prosthesis must be explanted and the tissue cleansed of pathological germs. Biofilm-forming bacterial strains can also be expected in open fractures. In order to kill the corresponding bacteria in this biofilm, 50–1,000 times higher levels of action of the antibiotic are necessary (13). Owing to toxic side effects of the antibiotics used, such levels of effect cannot be reached. Furthermore, reduced vascularity in chronic osteomyelitis significantly influences the effectiveness of systemic antibiotics (14–16). The focus of infection in the bone is surrounded by a connective tissue cuff made of avascular bone and thickened periosteum, muscle tissue and subcutaneous connective tissue. The avascularity of this scar tissue significantly reduces the effectiveness of systemically applied antibiotics (14–16). Infectious circulation deficits exacerbate this process.

As a result of this, another approach, additional local antibiotic treatment, which is applied to carrier materials or liquid, is needed. These locally effective antibiotic carriers also have disadvantages. Solids such as PMMA-ALC are common in the fact that they are not biodegradable and therefore remain in the body as a foreign substance or lead to the need for further operations to remove the material. This is a disadvantage for both the patient and the health system. These follow-up operations represent a mental burden for the patient, are considered a risk for wound infections and are both costly and time-consuming. Like any foreign body, the introduced PMMA-ALC is susceptible to the colonization of biofilm-forming bacterial strains (9). The resulting biofilm complicates the therapy; often the only option is the extraction of the foreign body. Another disadvantage of the non-absorbable materials for local antibiotic application is their unfavorable release kinetics (17, 18) and an incomplete elution of the drug (19, 20). Cierny et al. (21) showed that the release rates of tobramycin and cefotaxime are only between 25 and 50%, respectively, when applied mixed in PMMA balls. Comparable results were provided by an *in vitro* study by Wilson et al. (22), which showed that after 28 days on nutrient medium, 50–75% of tobramycin and cefotaxime was still detectable in the PMMA ball chains and so only 25–50% of the active ingredients were released from the PMMA ball chains. According to a study by DiCiccio et al. (20), the release rate even drops to 6–12% when the antibiotic in bone cement was formulated for prosthetic anchoring. The release rate of gentamycin dissolved in PMMA-ALC in intramedullary nails coated with PMMA-ALC in studies by Metsemakers et al. (23) and Hake et al. (24) lies between 4 and 17%. It can also be assumed that after an initial very high local level of action, rapid elimination of the antibiotic takes place and it is then present for a long time in subtherapeutic concentrations (25, 26). In order to compensate for the rapid degradation or defective elution of the active ingredients, no excessive increase in the active ingredients may be made, otherwise toxic concentrations can be achieved (27, 28).

The search for alternatives to local antibiotic therapy with non-degradable solids as carrier materials for the introduction of antibiotics shifted the focus to biodegradable substances. The advantage of these substances is that, due to their good biocompatibility and degradability by the body, only minor foreign body reactions are to be expected and thus do not further support an existing infection. Gels, powders, sponges and calcium sulfate are currently used in various formulations. In the search for other carrier materials, substances such as vitamin D3 and bioactive glass are the subject of current research (4).

The study presented here is a new concept for the local use of antibiotics. The fixation of antibiotics in the wound area by means of fibrin glue is an innovative and as yet little studied approach. The planned determination of the residence time and concentration of antibiotics in the wound area makes it possible to estimate whether the local bioavailability of the applied antibiotics can be improved by combining them with fibrin glue. Improved bioavailability should allow for more efficient antibiotic efficacy. Thus, in this study, we investigate whether the combination with fibrin glue slows down the absorption of the antibiotic. For this purpose, the local antibiotic concentration in the wound area is compared with the antibiotic concentration in the subcutaneous adjacent muscle tissue by means of mass spectrometry methods. In the long-term, we hope that this approach will be used to develop an improved method for the local application of antibiotics, which will directly benefit the patient's well-being. Since the individual components (fibrin glue and antibiotics) are clinically approved and established, we believe that a therapy that integrates both components has a good chance of realization.

The use of fibrin glue is already established and indicated in surgery if, in surgical procedures, hemostasis by manual pressure, ligatures or electrocauterization were ineffective or impractical (29, 30). Surface bleeding, for example, in diffuse bleeding of the liver or maxillary sinus, can be treated with fibrin glue (31, 32). The use of fibrin glue may be superior to electrocauterization, as electrocauterization leads to tissue necrosis which can increase the likelihood of infection later on (30, 33). Fibrin glue, on the other hand, offers the advantage of not causing necrosis, being large-scale and on an uneven surface and enabling both simple and cost-effective application. The handler needs both hands for the spraying process, in which compressed air from a hose distributes the contents of the prepared syringes filled with antibiotic and fibrin glue flatly and evenly on the wound bed. The spraying device is clinically established for this use (34, 35). The close contact of the fibrin glue to the wound bed and the retention of the antibiotic over a long period of time makes fibrin glue a suitable carrier for local antibiotic therapy (36, 37). The delivery of the antibiotic to the surrounding tissue is carried out either by dissolving the fibrin or by diffusion of the antibiotic by the fibrin matrix, depending on the antibiotic applied and the ratio of the antibiotic to the fibrin glue (4). However, the active concentration persists for several days above the minimum inhibitory concentration of common pathogens of wound infections (38–41). Since fibrin is a biological molecule found in humans naturally, no, or minimal immune reactions are to be expected. For the same reason, the use of fibrin glue does not interfere with wound healing mechanisms. The applied fibrin is degraded by the fibrinolytic system. At the same time, new bone matrix can be created, which gradually replenishes the wound defect. This can remove the need for a bony reconstruction (36). Since the components of the fibrin glue are broken down by the fibrinolytic system, a second intervention in the sense of material removal is not required (36, 42).

In the present work, it could be shown that stable colistin tissue concentrations can be achieved by fixation with fibrin glue. After 1 h, the median concentration of colistin with simultaneous spraying with fibrin glue stays above the laboratory technical upper detection limit of 185.67 ng per milligram tissue sample. In the corresponding comparison groups, the median concentration of colistin was only 89.45 ng per milligram tissue sample (consecutive spraying, after 1 h) or 60.95 ng per milligram tissue sample (only fibrin fixation, after 1 h) and thus significantly lower (Figure 1A). With simultaneous spraying and 2 h of duration of action, the median was still 61.19 ng per milligram of tissue, whereas the colistin concentration with consecutive spraying decreased after 2 h (47.47 ng/mg) and without fibrin fixation after 2 h (41.84 ng/mg) (Figure 1B). After 4 h, 37.00 ng per milligram of tissue remained detectable with simultaneous spraying, whereas the colistin concentration with consecutive spraying after 4 h (19.03 ng/mg) and without fibrin fixation after 4 h (9.95 ng/mg) also decreased. In the group without fibrin fixation after 4 h, five of the seven measured values were below the laboratory detection limit of 9.95 ng per milligram tissue sample (Figure 1C). The detected stable concentration of the antibiotic agent ensures a locally effective, bactericidal effect. Looking at the time course, there is a superiority of simultaneous spraying over consecutive spraying and spraying without fibrin fixation as shown in Figure 2. Whilst in the consecutive spraying group a significantly higher median concentration compared to the colistin only group could only be detected after 1 h, a significantly higher median concentration remains detectable after simultaneous spraying after 4 h, compared to the colistin only group. Thus, simultaneous spraying with colistin and fibrin glue seems to be the best application method to ensure this effect.

## Limitations of the Study

The present study compared the concentrations 1, 2, and 4 h after spraying the wound. A longer observation period can provide further insights into the dynamics of local antibiotic concentrations but cannot be realized due to the animal model. A wound model with bacterial colonies and their concentration development after the described spraying could also provide interesting insights with regard to the development of a solid therapy standard. Analogous to the soft tissue model of this study, a bone model with the same methodology is desirable to study the influence of local application of antibiotic and its fixation by means of fibrin on the osteomyelitis model.

## Conclusion

In exhausted treatment options, spraying a wound with colistin and fixing it by means of fibrin glue is an additional cure to the surgical debridement and systemic focused antibiotic therapy. The development of this spraying fixation method of antibiotics as an additional pillar of combined systemic and local antibiotic therapy can meaningfully expand the current range of treatments. The implementation of a fixed therapy algorithm for the treatment of SSTI seems reasonable. Even in chronic osteomyelitis, the local application of colistin and its fixation with fibrin glue might be a useful therapy option.

## Data Availability Statement

The raw data supporting the conclusions of this article will be made available on request from the corresponding author/s.

## Ethics Statement

The animal study was reviewed and approved by Darmstadt Regional Council (Project Number Gen. FK/1133).

## Author Contributions

RV and MJ performed the animal experiments. PW and RG performed the measurements. IM, DH, JF, and MJ were involved in planning and supervised the work. FD and DH processed the experimental data and designed the figures. IM and JF aided in interpreting the results. IM, JF, DH, RV, FD, and MJ worked on the manuscript. All authors discussed the results and commented on the manuscript.

## Conflict of Interest

The authors declare that the research was conducted in the absence of any commercial or financial relationships that could be construed as a potential conflict of interest.

## Publisher's Note

All claims expressed in this article are solely those of the authors and do not necessarily represent those of their affiliated organizations, or those of the publisher, the editors and the reviewers. Any product that may be evaluated in this article, or claim that may be made by its manufacturer, is not guaranteed or endorsed by the publisher.
